# Pharmacometabolomics by NMR in Oncology: A Systematic Review

**DOI:** 10.3390/ph14101015

**Published:** 2021-10-02

**Authors:** Nuria Gómez-Cebrián, Pedro Vázquez Ferreiro, Francisco Javier Carrera Hueso, José Luis Poveda Andrés, Leonor Puchades-Carrasco, Antonio Pineda-Lucena

**Affiliations:** 1 Drug Discovery Unit, Instituto de Investigación Sanitaria La Fe, 46026 Valencia, Spain; nuria_gomez@iislafe.es; 2 Ophthalmology Department, Hospital Virxen da Xunqueria, 15270 A Coruña, Spain; pedro.vazquez.ferreiro@sergas.es; 3 Pharmacy Department, Hospital Universitario La Plana, 12004 Castellón, Spain; carrera_fra@gva.es; 4 Pharmacy Department, Hospital Universitario y Politécnico La Fe, 46026 Valencia, Spain; poveda_joseand@gva.es; 5 Molecular Therapeutics Program, Centro de Investigación Médica Aplicada, 31008 Navarra, Spain

**Keywords:** pharmacometabolomics, nuclear magnetic resonance, drug response, personalized medicine, metabolism

## Abstract

Pharmacometabolomics (PMx) studies aim to predict individual differences in treatment response and in the development of adverse effects associated with specific drug treatments. Overall, these studies inform us about how individuals will respond to a drug treatment based on their metabolic profiles obtained before, during, or after the therapeutic intervention. In the era of precision medicine, metabolic profiles hold great potential to guide patient selection and stratification in clinical trials, with a focus on improving drug efficacy and safety. Metabolomics is closely related to the phenotype as alterations in metabolism reflect changes in the preceding cascade of genomics, transcriptomics, and proteomics changes, thus providing a significant advance over other omics approaches. Nuclear Magnetic Resonance (NMR) is one of the most widely used analytical platforms in metabolomics studies. In fact, since the introduction of PMx studies in 2006, the number of NMR-based PMx studies has been continuously growing and has provided novel insights into the specific metabolic changes associated with different mechanisms of action and/or toxic effects. This review presents an up-to-date summary of NMR-based PMx studies performed over the last 10 years. Our main objective is to discuss the experimental approaches used for the characterization of the metabolic changes associated with specific therapeutic interventions, the most relevant results obtained so far, and some of the remaining challenges in this area.

## 1. Introduction

Precision or personalized medicine aims to select, based on the characteristics of a patient, the most appropriate drug treatment for a particular disease. The ultimate goal in this area is to improve treatment efficacy and reduce the number of adverse effects [1,2]. However, this approach is challenging as patient responses to treatment can be very different [3]. In this context, pharmacogenomics (PGx) emerged as a promising approach for studying the influence of the specific individual’s genomic background on the response to drug treatment [4,5,6]. For certain drugs or drug classes, genetic factors have been shown to have the most important influence on drug treatment outcomes [7]. In fact, it has been reported that genetic traits account for 20–40% of the intra-patient differences associated with drug metabolism and response [8]. However, there exist other factors influencing drug response, including age, sex, disease, environmental factors, diet, and drug interactions [9,10]. Thus, although PGx approaches have facilitated the identification of many associations between genome alterations and changes in drug metabolism or response, they are somewhat limited as environmental or other contextual factors (i.e., ethnicity, diet, age, weight, gut microbiota, etc.) are not considered [11,12,13]. In this scenario, pharmacometabolomics (PMx) could represent a valuable alternative, or complementary, approach to PGx.

PMx, introduced in 2006 by Clayton et al. [14], focuses on predicting individual responses to drug treatments (i.e., toxicity and efficacy) based on the characterization of their metabolic fingerprints before the intervention [14]. The metabolic profile of a biological sample can be strongly influenced, from a quantitative and qualitative perspective, by a pathological condition or the presence of a specific drug [15]. The metabolome represents the final step of the omics cascade and can offer an accurate description of the pathophysiological status of an individual. Metabolomics provides information on metabolic changes induced by both environmental and genomic factors, therefore reflecting a more complete description of the molecular alterations associated with drug response than genomics [16]. This approach enables the identification of specific alterations in metabolites levels and pathways that characterize particular metabolic phenotypes associated with the specific patient’s response to a drug treatment [13,17]. PMx studies represent a promising approach for gaining a deeper insight into the molecular mechanisms that determine inter-patient variability in drug response [14,18,19,20]. Using this strategy, it is possible to identify metabolic biomarkers that could help in predicting individual drug effects and increasing efficacy in drug treatments. Since its introduction [14], the number of PMx studies has greatly increased, especially in the last decade. Patient metabolic profiles are frequently characterized using either the Nuclear Magnetic Resonance (NMR) or the Mass Spectrometry (MS) techniques, each of them exhibiting their own advantages and disadvantages. However, the high reproducibility, in addition to the non-destructive nature of the NMR-based approaches, presents a major advantage in these studies [21]. This review focuses on the analysis of the results derived from the NMR-based PMx studies performed over the last ten years.

## 2. Methods

### 2.1. Search Strategy

A systematic search was conducted on PubMed, Web of Science, and EMBASE databases for published NMR-based PMx studies, using the following terms: “(Pharmacometabolomics OR (Pharmaco OR Drug OR treatment OR response)) AND (Metabolomics) AND (Nuclear Magnetic Resonance OR NMR)”. In addition, the “Pharmacometabolomics” term was also introduced in the clinicaltrials.gov database to look for clinical studies using this experimental approach. Duplicates were removed and only the original articles written in English and published between January 2011 and June 2021 were selected for further review.

### 2.2. Selection Criteria

All selected publications were screened following standard protocols [22] and reviewed based on the pre-defined selection criteria. An additional filtering process based on the presence of the key terms “NMR AND (predict OR response OR effect OR pharmacometab) AND (patient OR human OR cell line)” in the title or abstract was also performed. Then, titles and abstracts of the selected publications were examined to evaluate their eligibility according to their relevance on the issue of interest in order to determine their inclusion in the review. Finally, the available full texts of selected articles were thoroughly reviewed. Additionally, principal investigators responsible for the PMx-related clinical trials identified in the clinicaltrials.gov database were contacted for further details on the experimental design of the studies in order to evaluate their potential inclusion in the review.

### 2.3. Data Extraction

The full-text articles of the final selected studies were reviewed in detail and different informative data were extracted, including disease, treatment, experimental design, sample type, time points for sample collection, research aim, NMR instrument and pulse sequence, data preprocessing, statistical analyses, etc.

## 3. Results

**Figure 1:**
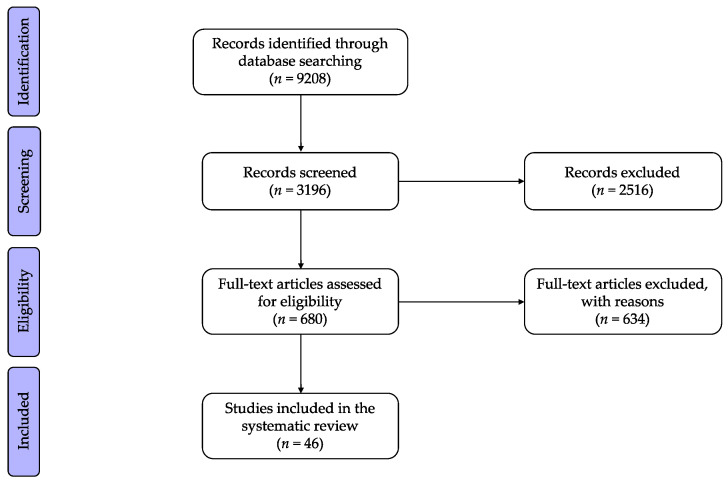
Preferred Reporting Items for Systematic Reviews and Meta-Analysis (PRISMA) flow diagram.

Out of a total of 9208 publications initially identified through the literature search (Figure 1), 3196 of them were published during the last ten years. After screening based on the pre-defined terms, 680 articles were considered eligible. A thorough review of the titles and abstracts of these articles led to a final selection of 46 studies matching the selection criteria previously described. Finally, the information included in the full-text publications of these 46 PMx studies was further analyzed for the purpose of this review.

### 3.1. Study Characteristics

#### 3.1.1. Sample Collection

Metabolomics analyses can be performed using multiple biological matrices. However, in the PMx studies included in this review the most frequently used biofluids were blood (i.e., serum, plasma, and platelets), followed by urine samples. Other biosamples, such as feces [23,24] or cells [25,26] were used in two studies, whereas saliva [27], culture medium [28], and tissue [29] were only collected in one of the studies. It should be noted that even though tissue and patient-derived cells can provide in situ information of the specific metabolic alterations due to a health condition or an external intervention [30], the access to these samples is highly dependent on the clinical practice. In metabolomics studies, it is recommended to follow specific standard operating procedures (SOPs) that harmonize processes associated with the quality of the biological samples: collection, processing, and storage [31,32]. Specific details regarding the protocols followed for sample collection were not included in most of the studies detailed in this review. It is of critical importance to ensure the quality of the samples used in PMx studies to avoid the introduction of additional, non-disease-related variations. Samples were stored at −80ºC until NMR analysis in the vast majority of the reviewed studies. Metabolomics studies based on the analysis of biofluids such as blood, urine, or saliva present obvious advantages due to their simple and less invasive collection. In particular, and despite the high variability in the number of samples, the number of participants included in the blood- or urine-related PMx studies was always significantly higher than in those based on the analysis of feces or tissue samples, most probably reflecting a much easier access and availability of these biofluids. Furthermore, studies relying on the analysis of patient-derived samples (i.e., biofluids, tissue, feces, etc.) included a larger number of samples compared with those focused on the analysis of commercially available cell lines. Overall, the number of samples included in the different PMx studies ranged from biological triplicates, in the case of cell cultures [28], to hundreds of patients, in the case of clinical trials [33].

#### 3.1.2. Study Design

Most of the reviewed PMx studies relied on the characterization of the metabolomics profiles of the patients, classified as “responders” or “non-responders”, to specific therapeutic interventions, using samples collected before treatment. Additionally, in 35 out of the 46 studies, patient samples were also collected at different timepoints after treatment. A number of studies also included the analysis of samples from a control group reflecting the metabolomics profile of healthy individuals. Overall, different experimental strategies are being explored for the evaluation of metabolic changes associated with drug response. For example, a very recent study evaluated metabolites produced by bacteria in ex vivo experiments. In particular, human stool samples were incubated in the presence of methotrexate to evaluate the association between the microbiome-driven metabolism of this drug and the clinical response to this therapeutic treatment [34].

#### 3.1.3. NMR Sample Preparation

The sample preparation in the different PMx studies followed the standard procedure used in most NMR-based metabolomics studies [31], consisting in the addition of a deuterated buffer to the blood and urine samples to adjust the pH and provide the necessary lock signal [31]. The pH adjustment turns especially relevant when samples, such as urine or saliva, that are particularly sensitive to inter-individual pH changes, are measured. In the PMx studies included in this review, the pH ranged from 6.8 to 7.4 for urine samples, whereas 7.4 was used for the saliva samples. The metabolomics profile in the only PMx study based on tissue samples was carried out using high-resolution magic angle spinning (HR-MAS) NMR spectroscopy [29]. Although this particular PMx study was performed using HR-MAS, a non-destructive method only requiring minimal sample preparation, some other metabolomics studies, such as those based on fecal samples, rely on a previous extraction of polar metabolites [35,36,37]. In general, the final percentage of deuterated water in samples not previously subjected to metabolite extraction (i.e., plasma, serum, saliva, urine, etc.) was approximately 10%, whereas polar extracts were usually lyophilized and resuspended in 100% D_2_O buffer. Furthermore, most of the studies relied on using sodium trimethylsilyl [2,2,3,3-^2^H_4_] propionate (TSP) as an internal standard, whereas 2,2,3,3-d_4_-3-(trimethylsilyl) propionic acid (TMSP) or 4,4-dimethyl-4-silapentane-1-sulfonic acid (DSS) were only used in a few studies. Other chemical compounds, such as tetramethylsilane (TMS) or calcium formate, were rarely used [38,39].

#### 3.1.4. NMR Spectra Acquisition

Operating frequencies ranging from 400 to 800 MHz were used in the different PMx studies, although 500 and 600 MHz spectrometers were the most frequently chosen. Furthermore, spectrometers equipped with a cryoprobe [24,26,40,41,42,43,44,45,46,47,48,49] were used in over 25% of the studies, and an automatic sample charger was only used in seven of them [24,26,48,50,51,52,53]. Spectrometer selection in the selected PMx studies does not appear to be associated with the type of sample or any other characteristic of the study. However, the selection of the NMR pulse sequence was heavily dependent on the sample type, as would be expected from the different nature of the biological matrices used in the studies. Thus, the Carr−Purcell−Meiboom−Gill (CPMG) [54] pulse sequence was preferentially used to acquire spectra from blood and tissue samples, whereas nuclear Overhauser effect spectroscopy (NOESY) [55] experiments were primarily selected for urine, fecal extracts, and saliva samples. Only one study relied on a different pulse sequence, Solvent-Optimized Gradient-Gradient Spectroscopy (SOGGY) [56], based on a previous excitation-sculpting template [57], to improve the water suppression and the solute sensitivity in the characterization of pancreatic cancer cells [26]. The CPMG pulse sequence, which includes a relaxation filter for larger molecules to facilitate the detection of small metabolites, was used for the measurement of samples containing lipids and proteins (i.e., serum, plasma, etc.) [31]. On the other hand, the NOESY experiment, which leads to spectra with improved baseline and water suppression [58], was the preferred choice for analyzing samples that do not usually contain large molecules (i.e., urine, polar extracts, cell culture media, etc.). The number of scans for acquiring the one-dimensional (1D) NMR spectra ranged from 16 to 256, independently of the sample type. The NMR metabolic profiles of biological samples are usually characterized by a high degree of signals overlap. In this context, the acquisition of two-dimensional (2D) NMR experiments (^1^H−^13^C Heteronuclear Single Quantum Correlation (HSQC), ^1^H−^1^H Total Correlation Spectroscopy (TOCSY), ^1^H-^1^H J-resolved spectroscopy (J-RES) [59], etc.) of representative samples greatly facilitates the assignment of the metabolites present in the biological samples [21,31]. The 2D NMR experiments were acquired in approximately 40% of the reviewed publications. Of note, only one of the reviewed studies included NMR-based stable isotope labelled approaches [26]. In addition, four of the selected studies integrated NMR and MS data [23,41,44,60], and a combination of PMx and PGx analyses was performed in one of the reviewed clinical trials [33]. The integrated analysis of data obtained through different analytical techniques and platforms offers very valuable information in these studies. In particular, the integration of NMR and MS data [23,61], as well as the application of multi-omics approaches, has shown a tremendous potential for the study of changes in metabolism [62,63].

#### 3.1.5. NMR Data Processing

Information on spectra processing was only partially, or not at all, detailed in most of the studies included in the review. Spectra phase and baseline correction were usually performed using TopSpin software (Bruker Biospin), although Chenomx (Chenomx) was chosen in other studies. Data binning was described as the first processing step in more than 50% of the studies. To this end, different software packages were used, including Amix (Bruker Biospin), Chenomx (Chenomx), MestreNova (Mestrelab Research S.L.), or NMRPRocFlow [64]. Blood and urine samples were generally binned into 0.04 ppm wide rectangular buckets, although smaller bucket widths (i.e., 0.005 or 0.002) were used in some of the selected PMx studies. An important issue in NMR-based metabolomics studies is the selection of the best compromise between the bucket size and the number of samples in the data set [65]. Even though very large bucket widths are not recommended as they decrease the resolution of the NMR spectra, an extreme reduction in the bucket width could significantly contribute to data overfitting as a result of the imbalance between the number of samples and the variables included in the analysis [66]. In general, most NMR-based metabolomics studies used a bucket width between 0.01 and 0.04 ppm, depending on the spectra complexity and the signal overlapping, for binning [29,43,67,68,69,70]. After binning, different normalization approaches were followed in most of the PMx studies. Although normalization details were not specified in all the studies, the normalization strategy was mainly dependent on the sample type. Overall, serum and plasma NMR data were preferentially normalized to total area [40,41,46,47,49,51,52,60,67,68,69,71,72,73,74,75,76], although probabilistic quotient normalization [26,48,77] and other normalization procedures, such as glucose [78] or internal standard normalization [39], were applied in some studies. For urine normalization, total area [42,67] and creatinine signal were the methods most frequently used [44,79,80]. Data normalization aims to make the data from all samples directly comparable and to reduce the effects of differences in sample dilution. Physiological normalization is especially relevant when analyzing biofluids such as urine where large differences in excreted volume, and hence in urinary concentrations, are found between patients. Different normalization approaches have been developed for the normalization of NMR-based metabolomics data. The most commonly used methods are normalization to total area and the use of endogenous stable metabolites (such as creatinine in urine) [81]. Additionally, data scaling is another important issue when analyzing NMR-based metabolomics data. In this context, pareto scaling seems to be the most sensible choice for NMR data scaling when the aim is data exploration through multivariate statistical approaches [82]. This particular approach was used in 15 of the 25 PMx studies including information on the method used for data scaling. In contrast to other scaling methods, such as unit variance, that often increase noise artefacts from spectral regions, pareto scaling increases the sensitivity and reduces noise [82]. Therefore, this scaling approach is often the method of choice for NMR spectra as the influence of small peaks is increased without amplifying uninformative variables [83].

#### 3.1.6. Metabolite Assignment

The analysis of the metabolomics data in all the PMx studies followed an untargeted approach. The assignment of metabolites was generally carried out using the information available through public databases (i.e., Human Metabolome Database (HMDB) [84,85] and Madison-Qingdao Metabolomics Consortium Database (MMCD) [86]), as well internal metabolic databases. Chenomx NMR Suite software [87] was used in 19 of the PMx studies for assignment purposes. The Chenomx NMR Suite is a commercially available software offering a large database of common biological and drug metabolite ^1^H-NMR data widely used for metabolomics analysis [88]. Although these two are probably the most extensive public metabolomics spectral databases, other open source compound libraries, such as the Biological Magnetic Resonance Data Bank (BMRB) [89] or InterSpin (RIKEN) [90] were used in some of the PMx studies. Additionally, there exist several metabolomics data repositories for submitting metabolomics datasets (i.e., MetaboLights [91], Metabolomic Repository Bordeaux [92], Metabolonote [93], etc.). Out of the 46 PMx studies selected in this review, only two of them [26,94] deposited their NMR dataset at the Metabolomics Workbench database [95], reflecting that the use of these repositories is still not very extensively used.

#### 3.1.7. Statistical Analysis

The most common strategy followed for statistical analysis of the data was multivariate analysis. First, unsupervised methods were used for the identification of inter-group variations, outliers, or sample clustering. Principal Component Analysis (PCA) [96] was the method of choice in most of the studies, although hierarchical clustering [97] was also used in other studies [26]. Then, supervised analysis methods, such as partial least square discriminant analysis (PLS-DA) [25,41,42,46,48,49,60,67,68,71,73,74,75,76,78,79,98,99] or orthogonal PLS-DA (OPLS-DA) [24,26,29,40,43,50,68,70,72,74,75,76,77,98,100,101,102] were pursued in the majority of the studies to evaluate the discriminatory potential of the metabolic profile between the groups of study. Herein, alternative supervised approaches were also followed, including multilevel Partial Least Square (mPLS) [27], Random Forest (RF) [45,47,53], K-nearest neighbors (kNN) [27], multivariate logistic regression analysis (MVLR) [78], or the GALGO R package [103], based on a genetic algorithm search procedure coupled to statistical modeling methods for supervised classification [42]. Furthermore, univariate analyses were performed to confirm the statistical significance of the metabolic changes identified based on the multivariate models. To that end, the Student T test or the Mann-Whitney U test were chosen for the mean comparison while Pearson or Spearman correlation analyses were followed for the evaluation of the potential correlations with continuous variables. Additionally, ROC curves were generated in 13 studies to internally validate the discriminatory power of their findings for predicting the response to treatment [24,26,41,44,45,46,47,52,70,74,77,78,99]. Nevertheless, none of the reviewed studies conducted an external validation to evaluate the relevance of their results in an independent set of samples. In general, SIMCA (Umetrics AB), SPSS (IBM Corp), Matlab (The MathWorks), PRISM (Graphpad), R software, and the online tool MetaboAnalyst [104,105] were the most frequently used software packages to perform the statistical analyses in the PMx studies. Two out of the four PMx studies carried out using a combination of two analytical approaches (i.e., NMR and MS) followed independent analyses for the data derived from each platform [41,44,60]. One of them performed an enrichment analysis based on the NMR data that facilitated the focus of the subsequent MS-based targeted analysis on the most significantly altered pathways [44]. In the other study, MS analyses were used to confirm the identity of specific metabolites involved in altered metabolic pathways [60]. The other two PMx studies performed an integrated multivariate analysis of both the MS- and the NMR-derived data. To that end, an additional block-scaling step was included to mitigate the effect of the difference in variances obtained in each analytical approach. In both cases, these analyses were performed using Matlab (The MathWorks) [23,41].

### 3.2. Therapeutic Areas and Treatments

**Figure 2:**
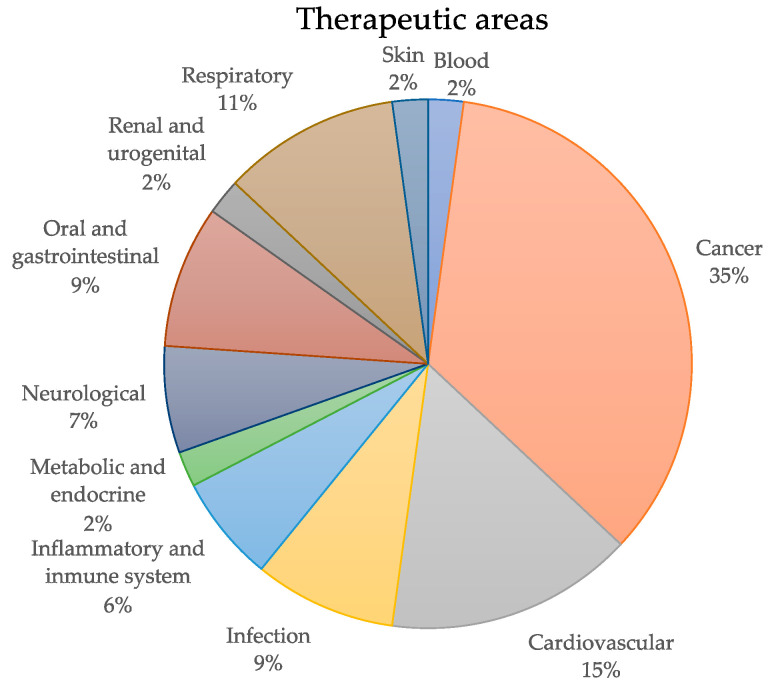
Pie chart displaying the classification of the NMR-based PMx studies based on the different health categories defined by the Health Research Classification System [106].

The 46 PMx studies included in this review can be classified into a total of 11 health categories, based on the Health Research Classification System (HRCS) [106] (Figure 2). More than one third of the reviewed articles focused on different oncological conditions [23,24,25,26,29,41,43,46,48,51,52,76,77,94,101,102]. Cardiovascular diseases, including coronary artery disease [49,70,74], hypertension [44], atrial fibrillation [78], myocardial infarction [107], and cardiotoxicity [28] were the second most explored health conditions. Five studies, associated with respiratory diseases, focused on the evaluation of treatments for chronic obstructive pulmonary disease (COPD) [68,73,98], acute lung injury [39], or COVID-19 [53]. Four other publications focused on the characterization of the metabolic profile associated with the treatment response in different infectious diseases, including septic shock [38], periodontitis [27] and HIV [45,47]. Other PMx studies (e.g., non-alcoholic fatty liver [80,99], alcohol use disorder [33], and drug-induced liver injury [79] treatments) were classified within the oral and gastrointestinal therapeutic area. Within the inflammatory and immune system area, rheumatoid arthritis was the subject of three different studies [42,71,72], and two publications focused on different neurological conditions, one on epilepsy [50,60] and the other on multiple sclerosis [69]. Finally, articles focused on β-thalassemia [75], vitamin D deficiency [67], nephrotic syndrome [40], and neonatal jaundice [100] were classified into the blood, metabolic and endocrine, renal and urogenital, and skin categories, respectively. Of note, out of the 46 PMx studies included in this review, only seven of them were associated with different clinical trials [29,33,38,43,80,94,107]. The most recent one, NCT03818191 [33], currently in the enrolling phase, pursues the combination of PGx and PMx strategies to identify biomarkers that could predict the response to the administration of acamprosate in patients with alcohol-use disorders.

Chemo- and immune-therapies were the therapeutic strategies most frequently evaluated in the PMx studies, particularly in the cancer category, but also in the cardiovascular, neurological, and respiratory diseases. For example, different studies evaluated changes in the metabolic profile of patients with breast cancer (BC) [24,41,94], pancreatic cancer (PC) [26,76], and head and neck squamous cell carcinoma (HNSCC) [101,102] receiving chemotherapy, while others focused on the effect of immune therapies in non-small-cell lung cancer (NSCLC) [23,52], BC [51], COVID-19 [53] and multiple sclerosis [69] patients. Different studies focused on the characterization of the metabolic profiles associated with different therapeutic strategies for the treatment of the same pathological condition. Thus, the effects of aspiring and clopidogrel, two anti-platelet agents, were evaluated in three different PMx studies related to coronary diseases [49,70,74]. Similarly, another study focused on the identification of biomarkers for predicting resistance to different drugs in epileptic patients [50]. Furthermore, the effects of bronchodilators [73,98] or antibiotics [68] were evaluated in PMx studies involving COPD patients. Finally, metabolic changes associated with two major treatment approaches were evaluated in patients suffering from rheumatoid arthritis, namely anti-tumor necrosis factor (TNF) inhibitors, including etanercept alone [72], or in combination with infliximab [42] and methotrexate [64].

### 3.3. Clinical Applications in Oncology

Oncology was the main therapeutic area explored in the NMR-based PMx studies included in this review. Sixteen PMx studies focused on different oncology conditions (Table 1). Therefore, this section will focus on the discussion of the most relevant results obtained in this area.

BC was by far the most frequently studied oncological disease [24,41,46,48,51,94]. Other studies analyzed the metabolic profile associated with PC [26,76], HNSCC [101,102], and NSCLC [23,52], and only one study referred to prostate cancer (PCa) [29], Hodgkin and non-Hodgkin lymphoma (HL/NHL) [25], hepatocellular carcinoma (HCC) [77], and multiple myeloma (MM) [43]. Overall, four different biological samples (i.e., serum, feces, cells, and tumor tissue) were used to evaluate metabolic changes in these studies. Most of the studies used serum samples, followed by feces [23,24] and cells [25,26], and tumor tissue, which was the biological matrix used in only one of the PMx studies [29].

#### 3.3.1. Breast Cancer

NMR-based PMx strategies for the evaluation of BC treatments were pursued in six studies with different objectives. Five of them focused on the identification of biomarkers that could contribute to the prediction of patient response to a specific treatment [24,41,46,48,51], and one aimed to characterize the metabolic profile associated with the development of adverse effects following paclitaxel treatment [94].

Jiang et al. [46] analyzed serum samples from 29 metastatic BC patients to characterize the pre-treatment metabolomics profile associated with the response to gemcitabine-carboplatin (GC) chemotherapy. By combining multivariate and univariate analyses, metabolic differences between clinically-benefited and non-benefited patients were identified. Furthermore, the relevance of the most altered serum metabolites for predicting the response to chemotherapy was evaluated using ROC curves. Based on this analysis, formate and acetate basal levels showed a high sensitivity (>0.8) and specificity (>0.8) for predicting treatment response. The authors suggested that the decreased formate and acetate levels observed in the non-responding patients could be reflecting the use of these metabolites as an alternative nutritional source to fulfill the energetic needs of highly proliferating cancer cells, which are more aggressive or resistant to therapy. Metabolic alterations capable of predicting the response to different neoadjuvant chemotherapy regimens in BC patients were also evaluated in a PMx study conducted by Wei et al. [41]. In this other study, the differences in the serum metabolic profile of 28 BC patients with complete (CR), partial (PR), or no-response (NR) to neoadjuvant chemotherapy (NAC), using a combination of NMR and liquid chromatography (LC)-MS metabolomics approaches, were characterized. A statistical model based on the analysis of the levels of three metabolites detected by the NMR (threonine, glutamine, and isoleucine) and one by LC-MS (linolenic acid) provided 100% selectivity and 80% sensitivity for the prediction of CR vs. NR patients. Changes in the serum metabolic profile of HER2-positive BC patients after treatment were also evaluated by Jobard et al. [51]. Samples from 79 patients receiving either trastazumab alone (*n* = 40) or a combination with everolimus (*n* = 39) were collected before, during, and after treatment administration. Everolimus is an inhibitor of the mammalian target of rapamycin (mTOR) and trastuzumab, a monoclonal antibody able to bind HER2, inhibits the proliferation of cells overexpressing HER2 [108]. Results showed that the combination (tratuzumab + everolimus) induced significant changes in the metabolism of patients that were not induced by trastuzumab alone. The BC patients treated with the combination therapy exhibited increased levels of lipids (the glycerol backbone of phosphoglycerides), triacylglycerides, lipoproteins (VLDL and LDL), and acetone and decreased levels of acetate, amino acids (alanine, histidine, lysine, phenylalanine, tyrosine, and valine), albumin lysyl, betaine, creatine, creatinine, acetoacetate, citrate, choline, glucose, glycerophosphocholine, myo-inositol, and methanol levels. Some of the metabolic changes detected in the serum metabolics profile of the BC patients were consistent with metabolic changes previously described in relation to mTOR inhibition [108,109,110,111,112,113,114,115]. Hence, although synergistic effect could not be completely excluded because the study did not include a subgroup of patients treated with everolimus alone, the metabolic signature observed for the combination treatment could most likely be reflecting mTOR inhibition.

The impact of NAC and other therapeutic approaches in the metabolic profile of BC patients has also been evaluated. Debik et al. [48] evaluated the metabolomics profiles of 118 primary BC patients (tissue, serum) receiving NAC alone, or a combination with bevacizumab, to identify potential changes associated with treatment response or patient prognosis. Results revealed significant alterations in the serum metabolites during treatment, particularly in a significant increase in lipid levels during NAC. Furthermore, specific metabolic changes, including higher levels of leucine, acetoacetate, and tri-hydroxybutyrate were observed in patients treated with bevacizumab. Interestingly, in this study tissue metabolic profiles exhibited a predictive potential for discriminating survivors from non-survivor patients in this study, while serum metabolite levels reflected the patient response to treatment. The patient response to NAC was also evaluated by Zidi et al. [24] using a strategy based on the analysis of metabolic alterations of BC patients before and after three chemotherapy cycles. Specifically, the fecal metabolomic profiles of six good- and two non-responder BC patients were characterized with a focus on the identification of potential candidate biomarkers that could predict the response to NAC. Multivariate supervised analyses showed that the treatment effect started to affect the fecal metabolome of patients after the second cycle of treatment. Interestingly, the levels of short chain fatty acids (SCFA), specific products of the gut microbiota, also exhibited a tendency to increase after the second cycle. Moreover, the good-responder patients showed specific metabolic changes after NAC, including higher levels of some amino acids, creatine, phenylacetate, 3-methylhistidine, histamine, ethanol, theophylline, and succinate when compared with the non-responder patients. These results suggest that changes in the fecal metabolic profile of BC patients could provide very relevant information on the biochemical changes associated with NAC.

In addition, a PMx study, carried out in the context of a clinical trial (NCT02338115), focused on the identification of serum metabolic alterations associated with the development of paclitaxel-induced peripheral neuropathy (PN) [94]. To this end, serum samples from 48 BC patients were collected before, during, and after treatment with paclitaxel. Using this strategy, the potential association between changes in the serum metabolic profile of patients and ΔCIPN8 scores, a parameter measuring primarily sensory neuropathy caused by paclitaxel, was evaluated. Inverse correlations between the pre-treatment levels of histidine, phenylalanine and threonine, and the maximum ΔCIPN8 were observed, suggesting that these amino acids could potentially predict PN severity in these patients. In fact, as indicated by Sun et al. [94], histidine is involved in the pathogenesis and inflammatory process of neuropathic pain [116,117,118]; phenylalanine precursors are implicated in the development of neurological conditions [119,120]; and threonine could cause glycine accumulation in the brain, affecting neurotransmitter balance [121]. This study highlights the enormous potential of PMx studies in the follow-up of BC patients.

#### 3.3.2. Pancreatic Cancer

PMx studies focused on PC have relied on different in vitro and in vivo models to characterize the metabolic changes associated with the response or resistance to therapeutic interventions. Gebregiworgis et al. investigated the potential of PMx to differentiate PC cells that respond or develop resistance to Gemcitabine treatment [26], information that could be useful in the clinical setting for monitoring a patient’s therapeutic response. In particular, the authors compared the metabolomics profile of wild-type (WT) and Gemcitabine-resistant (GemR) PC cell lines before and after treatment with Gemcitabine. Analysis of the metabolomics profile after treatment in the two experimental models (WT and GemR) allowed the identification of unique metabolic changes differentiating the response, or the acquired resistance, to gemcitabine. Overall, the metabolic profile associated with gemcitabine-resistance was the major feature discriminating between the groups of study. Specific alterations in the metabolism of GemR cells were further evaluated by combining stable-isotope labeling experiments using ^13^C_6_-glucose. Based on these studies, it was concluded that, in GemR cells, glucose is primarily derived for nucleotide synthesis to compensate gemcitabine activity; whereas in WT cells, glucose is primarily directed into glycolysis after treatment with Gemcitabine. These findings are in agreement with previous results reporting that Gemcitabine efficacy is influenced by the nucleotide cellular pool [122] and that deoxycytidine triphosphate acts as a competitive-inhibitor of Gemcitabine [123]. A different study, conducted by Wei et al., has also evaluated the therapeutic effects of Cucurmosin, as an alternative to Gemcitabine for PC treatment, by examining its impact on serum metabolism [76]. Differences in the serum metabolomics profile after treatment were evaluated in a subcutaneous xenograft mouse model of PC. The results showed that whilst the PC mice showed specific metabolic changes when compared with the control mice, both drugs induced similar metabolic effects in the in vivo PC model. Additional studies would be required to explore the significance of these changes in disease progression and the response to treatment.

#### 3.3.3. Head and Neck Squamous Cell Carcinoma

Treatment response and adverse effects, associated with different therapeutic interventions, have been evaluated in different NMR-based PMx studies focused on HNSCC patients. A first study pursued the NMR characterization of the serum metabolic profile of HNSCC patients following radio- and/or chemotherapy to identify metabolic alterations that could predict weight loss and induced-toxicity risk [101]. Serum samples from 170 patients undergoing radio- and chemotherapy (RT/CHRT) were weekly collected before, during, and after treatment. The authors identified a group of three ketone bodies (3-hydroxybutyrate (3HB), acetone and acetoacetate) able to identify patients at high risk of weight loss. Particularly, 3HB was found to be a sensitive biomarker for the identification of patients at higher risk of >10% weight loss during RT/CHRT treatment. In a more recent study, the serum metabolic profile of 53 locally-advanced HNSCC patients was also analyzed to identify biomarkers able to differentiate responder from non-responder patients [102]. The analysis of the metabolomics profiles revealed an association between the response to induction chemotherapy (iCHT) and increased serum lipids, accompanied by a simultaneous decrease in alanine, glucose, and N-acetyl-glycoprotein (NAG) levels. These metabolic changes were initially associated with the regression of the primary tumor in males. However, an in-depth analysis of the data suggested that gender-related metabolic differences could be explained by elevated pre-treatment levels of glucose and alanine and/or a higher initial tumor stage found in the male patients enrolled in the study [102].

#### 3.3.4. Non-Small-Cell Lung Cancer

Two PMx studies have evaluated metabolic alterations associated with the immunotherapy response in NSCLC patients, using serum and feces. Ghini et al. evaluated the serum metabolomics profile of NSCLC patients before treatment with the immune checkpoint inhibitors Nivolumab and Pembrolizumab [52]. The classification model derived from this analysis allowed the prediction of individual outcomes with >80% accuracy, and the results showed that the serum metabolic fingerprints able to discriminate responder from non-responder patients were similar for both treatments. Another study carried out by Botticelli et al. has been able to identify metabolites specifically associated with the Nivolumab response using a strategy based on the combined analysis (NMR, MS) of the fecal metabolic profile of nine NSCLC patients after Nivolumab [23]. Higher levels of 2-Pentanone (ketone) and tridecane (alkane) were significantly associated with early disease progression in this study, whereas higher levels of SCFAs (i.e., propionate, butyrate), lysine, and nicotinic acid were significantly associated with a better treatment response. These preliminary data suggest a potential role of gut microbiota metabolic alternations in regulating the response to immunotherapy.

#### 3.3.5. Prostate Cancer

So far, only one PMx study, based on HR-MAS NMR spectroscopy, has been carried out with a focus on PCa patients. In particular, Madhu et al. evaluated the metabolic changes after treatment with Degarelix, a gonadotrophin-releasing hormone blocker used to treat advanced PCa by decreasing serum androgen levels, in intact prostate tissue [29]. To this end, benign and tumor tissue samples were collected from 13 PCa patients participating in two different clinical trials (NCT01852864 and NCT00967889 for treated and untreated patients, respectively). The results of the NMR and the statistical data revealed that lactate, alanine, and choline levels were significantly increased in high-grade PCa tumors compared with benign samples. Furthermore, the Degarelix treatment resulted in significant decreases in lactate and choline levels in tumor samples, whereas these changes were not observed in benign prostate tissues. The results from this study suggest that it could be possible to monitor the effects of physical or chemical castration in PCa patients based on their metabolomics profile changes.

#### 3.3.6. Hodgkin and Non-Hodgkin Lymphoma

Peripheral blood stem cells from HL/NHL patients, collected before hematopoietic cell transplantation (HCT), were metabolically examined by Cano et al. to identify patients with a higher predisposition for developing therapy-related myelodysplasia syndrome or acute myeloid leukemia (t-MDS/AML) [25]. Patients were classified based on the occurrence of t-MDS or AML within 5 years after autologous hematopoietic cell transplantation (aHCT). Comparison of the metabolite levels between patients developing (*n* = 6) and not-developing (*n* = 6) t-MDS/AML resulted in the identification of alterations in alanine and aspartate metabolism; glyoxylate and dicarboxylate metabolism; phenylalanine metabolism; the citrate acid cycle; and aminoacyl-t-RNA biosynthesis. The authors suggested that these metabolic dysfunctions would result in a decreased ability of cells to detoxify reactive oxygen species (ROS) derived from therapy, leading to DNA mutations that could predispose patients for the development of t-MDS.

#### 3.3.7. Hepatocellular Carcinoma

The serum metabolic profile of 120 HCC patients was analyzed by Goossens et al. to identify the metabolic changes associated with disease recurrence and the radiofrequency ablation (RFA) response in these patients [77]. Although no significant findings were identified for defining a predictive signature of HCC recurrency, the serum metabolic profile of patients analyzed before treatment showed significant differences depending on whether the liver disease had a viral or a non-viral etiology. Moreover, several metabolic alterations were found when comparing serum samples at different time points. Thus, the RFA response was correlated with higher levels of lactate, glutamine, and 3-phenylpropionate, as well as lower levels of isoleucine, phosphatidylcholine, and glycerophosphocholine. Furthermore, some other metabolites, including lipids, aspartate, choline, and glucose experienced different alterations four months after RFA in viral and non-viral cirrhosis patients, reflecting different metabolic patterns of evolution after RFA depending on the etiology of the cirrhosis.

#### 3.3.8. Multiple Myeloma

Serum samples from healthy individuals and MM patients were collected at the time of diagnosis and after complete remission and metabolically characterized to obtain clinically relevant information for the management of this oncological condition [43]. This PMx study relied on the analysis of samples from two different clinical trials (NCT00461747, NCT00443235). Specific metabolic changes were identified in MM patients at the time of diagnosis, but also after complete remission of the disease. A comparison of the metabolic profiles obtained for the different groups of the study resulted in the identification of metabolic alterations (i.e., glutamine, cholesterol, and lysine) observed at the MM diagnosis that exhibited an opposite trend in MM patients upon responding to treatment. This behavior would explain why MM patients after complete remission exhibited a more similar metabolic profile to that of healthy individuals. Interestingly, it was also found that some other metabolic alterations associated with the disease (i.e., 3-hydroxybutyrate, arginine, and acetate) were not reversed after achieving complete remission and could potentially play a role in MM relapse.

## 4. Conclusions and Future Perspective

It is becoming increasingly important to accurately select the best therapeutical strategy for a specific health condition in order to maximize the therapeutic benefit of a specific group of patients. PMx relies on the characterization of patient metabolic profiles to better understand the molecular mechanisms underlying drug administration, predict patient drug response, and identify biomarkers associated with drug toxicity. Therefore, PMx represents a powerful experimental strategy to gather information on drug safety, toxicity, or metabolism, because it involves the evaluation of a wide variety of factors, including specific genetic traits and environmental parameters. In this context, PMx studies, based on the non-invasive evaluation of metabolic changes, could improve the current landscape of precision medicine by providing more accurate and specific predictions on drug efficacy and safety. This review underlines the tremendous potential of these approaches for the evaluation and prediction of treatment efficacy and safety in different oncological conditions.

However, standardized protocols for optimal sample preparation [124]; the need of sensitive, specific and reproducible analytical approaches [125]; and the importance of accurate data processing for reliable statistical analysis [126] are still under development in this research area. Furthermore, other factors, often underestimated, that could have a major impact on metabolomics analyses include sample collection, processing, or storage [127,128]. In fact, different protocols for sample preparation and NMR-data acquisition and pretreatment were followed, even for the same sample types in the PMx studies included in this review. Therefore, the implementation of standard operating procedures (SOPs) could contribute to ensure reproducibility across research centers and biobanks [129]. This strategy could also facilitate the development of sufficiently well-powered datasets for producing accurate and robust findings that could potentially be translated to the clinical setting. Only a few of the PMx studies relied on using different analytical techniques (e.g., NMR and MS) or platforms (e.g., metabolomics, genomics, proteomics, etc.). In this context, the integrated analysis of the data from different experimental approaches to the characterization of treatment effects in patient samples on future PMx studies could enormously benefit the personalized medicine field and further improve the treatment selection for patients. Additionally, an in-depth characterization of the metabolic changes, based on the analysis of different in vivo and in vitro approaches, could also provide a better understanding of the biological mechanisms underlying metabolic changes. Overcoming such challenges is essential to discovering sensitive and specific biomarkers that could be informative on drug metabolism, safety, efficacy, and response.

## Figures and Tables

**Table 1:** Overview of the PMx studies focused on the oncology area.

Disease	Treatment	Experimental Design	Sample	Sample Collection	Research Aim	NMR Instrument	Pulse Sequences	Reference
BC	GC chemotherapy	29 (1 CR, 13 PR, 8 SD, 7 PD)	Serum	Before treatment	Prediction of treatment response	800 MHz	1D: CPMG 2D: COSY, HMBC, HSQC, J-RES, TOCSY	[46]
BC	NAC	28 (8 CR, 14 PR and 6 NR)	Serum	Before treatment	Prediction of treatment response	500 MHz	CPMG	[41]
HER2+ BC	T/T+E	79 (40 T, 39 T+E)	Serum	Before, during, and after treatment	Evaluation of treatment impact	800 MHz	1D: CPMG, NOESY 2D: HSQC, J-RES, TOCSY	[51]
BC	NAC/ NAC + Bev	118 (58 NAC, 60 NAC + Bev)	Tissue and serum	Before and during treatment, and 6 weeks after surgery	Evaluation of treatment impact Prediction of patient prognosis	600 MHz	CPMG	[48]
BC	NAC	8 (6 good, 2 non-responders)	Feces	Before and 20 days after each chemotherapy cycle	Evaluation of treatment impact Prediction of treatment response	600 MHz	1D: NOESY 2D: COSY, HSQC, TOCSY	[24]
BC	Paclitaxel	48	Blood	Before, during, and after treatment	Prediction of treatment adverse effects	500 MHz	1D-1H-NMR	[94]
PC	Gemcitabine	10 replicates	Cell lines	Before and after treatment	Biomarkers of treatment resistance and response	500 MHz	1D-SOGGY 2D: HSQC	[26]
PC	Gemcitabine/CUS	50 (12 control, 9 PC, 10 CUS-high, 10 CUS-low, 9 gemcitabine)	Serum from xenografts	33 days after treatment	Evaluation of treatment impact	600 MHz	CPMG	[76]
HNSCC	Radio-/Chemo-therapy	170	Serum	Weekly, from the day before to the week after treatment	Prediction of treatment adverse effects	400 MHz	1D: CPMG, DIFF, NOESY 2D: J-RES	[101]
HNSCC	Induction chemotherapy	53	Serum	Before and after treatment	Prediction of treatment response	400 MHz	1D: CPMG, DIFF, NOESY 2D: J-RES	[102]
NSCLC	Nivolumab/Pembrolizumab	50 (34 nivolumab, 19 pembrolizumab)	Serum	Before treatment	Prediction of treatment response	600 MHz	CPMG, DIFF NOESY	[52]
NSCLC	Nivolumab	9 (4 EP, 5 LR)	Feces	After treatment	Prediction of treatment response	400 MHz	2D: HSQC, TOCSY	[23]
PCa	Degarelix	13 (10 benign, 7 PCa untreated, 6 PCa treated)	Tissue	7 days after treatment	Evaluation of treatment impact	600 MHz	CPMG	[29]
HL/NHL	High dose therapy	12 (6 t-MDS/AML, 6 no t-MDS/AML)	Peripheral blood stem cells	Before aHCT	Evaluation of metabolic changes associated to adverse effects	600MHz	1D-1H-NMR	[25]
HCC	RFA	120 (59 viral, 61 Non-viral cirrhosis)	Serum	Before and after treatment	Prediction of treatment response	500 MHz	1D: CPMG, NOESY 2D: J-RES, TOCSY	[77]
MM	Chemotherapy	81 (31 control, 27 diagnosed, 23 remission)	Serum	Before and after treatment	Evaluation of treatment impact	600 MHz	1D: CPMG, NOESY 2D: HSQC, J-RES, TOCSY	[43]

aHCT: autologous hematopoietic cell transplantation; Bev: bevacizumab; BC: breast cancer; COSY: ^1^H-^1^H correlation spectroscopy; CPMG: Carr-Purcell-Meiboom-Gill; CR: complete response; CUS: cucurmosin; DIFF: diffusion edited; E: everolimus; EP: early progressors; GC: gemcitabine-carboplatin; HCC: hepatocellular carcinoma; HER2: Human Epidermal growth factor Receptor type-2; HL: Hodgkin lymphoma; HMBC: ^1^H-^13^C heteronuclear multiple bond correlation spectroscopy; HNSCC: head and neck squamous cell carcinoma; HSQC: ^1^H-^13^C heteronuclear single quantum correlation spectroscopy; J-RES: J-resolved spectroscopy; LR: long responders; MM: Multiple myeloma; NAC: neoadjuvant chemotherapy; NHL: non-Hodgkin lymphoma; NOESY: Nuclear Overhauser effect spectroscopy; NR: no-response; NSCLC: non-small-cell lung cancer; PC: pancreatic cancer; PCa: prostate cancer; PD: progressive disease; PR: partial response; RAF: radiofrequency ablation; SD: stable disease; SOGGY: Solvent-Optimized Gradient-Gradient Spectroscopy; T: trastuzumab; t-MDS/AML: therapy-related myelodysplasia syndrome or acute myeloid leukemia; TOCSY: ^1^H-^1^H total correlation spectroscopy.

## Data Availability

Data sharing not applicable.
